# Fully integrated multi-optoelectronic synthesizer for THz pumping source in wireless communications with rich backup redundancy and wide tuning range

**DOI:** 10.1038/srep29084

**Published:** 2016-07-06

**Authors:** Junjie Xu, Lianping Hou, Qiufang Deng, Liangshun Han, Song Liang, John H. Marsh, Hongliang Zhu

**Affiliations:** 1 Institute of Semiconductors, Chinese Academy of Sciences, No. A35, East Qinghua Road, Haidian District, Beijing 100083, P.R. China; 2 School of Engineering, University of Glasgow, Glasgow, G12 8LT, UK

## Abstract

We report a monolithic photonic integrated circuit (PIC) for THz communication applications. The PIC generates up to 4 optical frequency lines which can be mixed in a separate device to generate THz radiation, and each of the optical lines can be modulated individually to encode data. Physically, the PIC comprises an array of wavelength tunable distributed feedback lasers each with its own electro-absorption modulator. The lasers are designed with a long cavity to operate with a narrow linewidth, typically <4 MHz. The light from the lasers is coupled via an multimode interference (MMI) coupler into a semiconductor optical amplifier (SOA). By appropriate selection and biasing of pairs of lasers, the optical beat signal can be tuned continuously over the range from 0.254 THz to 2.723 THz. The EAM of each channel enables signal leveling balanced between the lasers and realizing data encoding, currently at data rates up to 6.5 Gb/s. The PIC is fabricated using regrowth-free techniques, making it economic for volume applications, such for use in data centers. The PIC also has a degree of redundancy, making it suitable for applications, such as inter-satellite communications, where high reliability is mandatory.

Wireless data traffic has increased dramatically in recent years–according to Edholm’s law of bandwidth, doubling every eighteen months–and is quickly approaching the capacity limit of present wired communication systems^1^. Current trends suggest that wireless Terabit-per-second links will become a reality within years. In addition to the traditional wireless bands, the THz band of frequencies between 0.1 THz and 10 THz will be needed to support these extremely high data rates^2^. The scarcity of the wireless spectrum and capacity limitations of current wireless systems will thus be alleviated^3^,^4^.

Terahertz sources based on semiconductor technologies have the advantage of a small form factor, which is necessary if THz wireless systems are to be used in computer and low-cost data applications, such as ultra-high speed on-chip communications or data storage^5^, and data center communications or satellite to satellite communications^6^. Recently, a tunable monolithic THz source has been reported using quantum cascade lasers^7^,^8^. Tunability over the range of 3.44~4.02 THz was demonstrated at room temperature^7^. Mode-beating of light from dual cascaded Distributed Feedback Lasers (DFBs) integrated with a phase section have also been designed to generate THz waves^9^, and a continuous tuning range from 0.3 THz to over 1.34 THz was demonstrated. Recently, a tuning range covering 0.15–3 THz has been realized using a two-section digital DFB laser diode^10^. Though these approaches allow tuning over a broad THz spectrum, extra modulators will be needed for signal encoding in communication applications, which will increase cost and complexity. Moreover, some applications require extreme reliability with adequate redundancy, but there owns no channel backup capability in these approaches.

Monolithically integrated sub-systems based on photonic integrated circuits (PICs) techniques have recently undergone a period of rapid development^11^,^12^,^13^,^14^,^15^, but PICs which are designed to generate THz carrier frequencies have not yet been explored in any depth. Studies of PIC-based approaches for wireless communication are few and have been limited to gigahertz frequencies^16^,^17^.

In our paper, we first present an indium phosphide-based PIC designed to generate an optical carrier modulated at a frequency in THz range–an optoelectronic THz frequency pumping source. Compared to a planar silicon platform^18^, DFBs, semiconductor optical amplifiers (SOAs) and electro-absorption modulators (EAMs) in particular can all be integrated monolithically in an InP PIC, avoiding the needs of demanding coupling or hybrid integration techniques, such as taper coupling or wafer bonding. Therefore, an InP-PIC offers more simplicity and better reliability. Continuous tuning from 0.254 THz to 2.723 THz can be realized by biasing pairs of DFBs at appropriate currents.

The PIC has an array of four DFB lasers (DFB1–DFB4) generating up to 4 optical frequencies. A THz signal can be produced by mixing the light from pairs of lasers through difference-frequency generation (DFG) in a nonlinear element such as a second harmonic generation (SHG) crystal or a detector photodiode (square law detector). For example, mixing light from DFB1 emitting with an optical frequency *f*_1_ with light at *f*_2_ from DFB2 generates a THz frequency at *f*_1_–*f*_2_. The DFG signal is highly sensitive to the linewidth and the noise level of each DFB, so each laser has a relatively long cavity (1.155 mm) to ensure a narrow linewidth, typically <4 MHz. Each of the four DFBs has its own EAM, utilizing the quantum-confined stark effect (QCSE) mechanism to balance the intensities between each two DFB lasers. These external modulators also enable data encoding while ensuring stable laser operation.

In this paper, we observe the THz modulation using an autocorrelator based on SHG. In future systems, the THz electromagnetic wave will be launched by detecting the light in a fast photodiode or photoconductive detector coupled to an antenna. Because of the design of the PIC, certain THz difference frequencies within the continuous tuning range can be accessed using more than one pair of lasers. This rich channel-backup redundancy may be important in applications where extreme reliability is required–for example in inter-satellite communications.

## Design and fabrication details of the PIC

**Figure 1:**
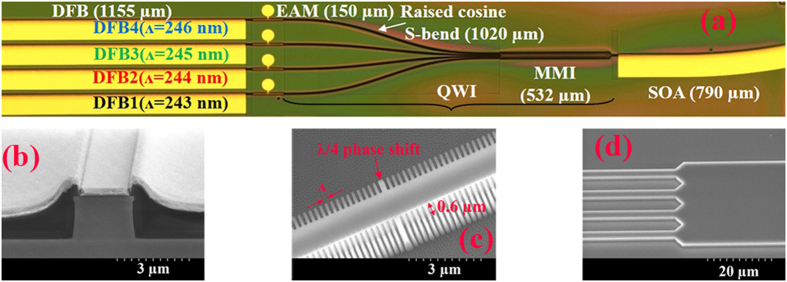
Device dimensions and scanning electron microscope (SEM) pictures of ridge waveguide, gratings, and MMI coupler. **(a)** Optical microscope picture of the overall laser array, **(b)** SEM picture of the cross section of the ridge waveguide, **(c)** first order side-wall gratings with 0.6 μm recess depth, **(d)** input to MMI coupler section.

An optical micrograph and the dimensions of the device are shown in Fig. 1(a), the fabrication processes being similar to those described in ref. 19. The epitaxial structure is the same as that described in ref. 20.

The separation between two adjacent lasers was set at 125 μm. The ridge width was 2.5 μm (Fig. 1(b)). The passive section, which includes the S-bend waveguides and the multimode interference (MMI) coupler, was blue-shifted by 100 nm using the quantum well intermixing technique. The raised cosine S-bends were 1020 μm-long. The MMI coupler was 30 μm-wide and 532 μm-long (Fig. 1(d)). The purpose of 790 μm-long SOA was to compensate the losses from the S-bend and the MMI coupling. The SOA was designed with a curved waveguide terminating at an angle of 10° relative to the normal direction of the facet to minimize facet reflectivity. Each of the DFB lasers had a total length of 1155 μm. The grating periods (*Λ*) were 243–246 nm for DFBl to DFB4 respectively, with corresponding Bragg wavelengths of *λ*_1_ (1550 nm), *λ*_2_ (1556.46 nm), *λ*_3_ (1562.84 nm), *λ*_4_ (1569.22 nm). The gratings were of first-order with a 50% duty cycle and formed by etching recesses of depth *d *= 0.6 μm into the sidewalls of the ridge waveguide of each of the DFB lasers, as shown in Fig. 1(c). A λ/4 phase shift was inserted in the center of the cavity of each laser (Fig. 1(c)) to ensure single longitudinal mode (SLM) operation. The coupling coefficient of the gratings, *κ*_0_, was measured to be approximately 80 cm^−1^ using the sub-threshold spectral fitting method. The design of the EAMs was based on the identical active layer (IAL) monolithic integration approach in which their epitaxial structure is the same as that of the lasers. The inherently wide gain spectrum exhibited by strained MQWs structures (~50 nm 3-dB gain bandwidth)^21^ allows the DFB laser wavelengths to be detuned to longer wavelengths than the excitonic absorption bandedge of the EAMs, allowing flexibility in the design of insertion losses and the modulation depths. These EAMs, which own a length of 150 μm, are settled to balance the power from the two lasers and for encoding digital data at 5 Gb/s with a clear open eye pattern.

### Characterization

**Figure 2:**
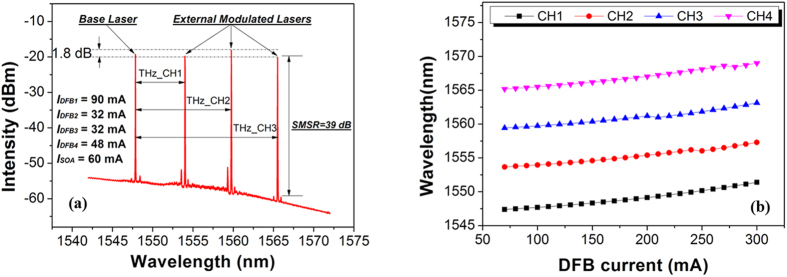
Three THz channels formed by four laser channels with individual tenability. **(a)** The optical spectrum with all four laser channels operating simultaneously; *I*_*DFB1*_ = 90 mA, *I*_*DFB2*_ = 32 mA, *I*_*DFB3*_* *= 32 mA, *I*_*DFB4*_ = 48 mA with *I*_SOA_ = 60 mA and the EAMs floating. **(b)** Tuning of the DFB lasing wavelengths with drive current, with *I*_*SOA*_ = 80 mA and at temperature of 20 °C.

The chip was mounted on a temperature controlled copper sink and all tests were performed at a temperature of 20 °C. The output spectrum with all four lasers operating simultaneously is presented in Fig. 2(a). Because of the *λ*/4 phase shift introduced in the DFB lasers, each of the lasers operated in a stable single longitudinal mode. As is indicated in Fig. 2(a), DFB1 was selected as the base laser with its external EAM left floating; the other lasers could be modulated via each of their EAMs, and up to three different communication channels with a THz carrier frequency could therefore be selected. By biasing EAMs at property voltages, the intensity of each laser could be controlled individually, the intensity difference between the two lasers can be kept less than 2 dB. At this stage, our emphasis is to analyze the tuning and redundancy frequency properties of the THz channels from our InP-PIC.

As shown in Fig. 2(b), each of the four DFBs in the array can be tuned in wavelength by changing its drive current. By linear fitting to the results, the tuning rate was measured to be 0.016 nm/mA. With the current held constant, the tuning coefficient with temperature is around 0.1 nm/K in the range −5–70 °C. Each DFB had a current tuning range of around 4 nm, with the largest wavelength difference between DFB1 and DFB4 being 21.61 nm. No mode hopping was observed during current tuning.

**Figure 3:**
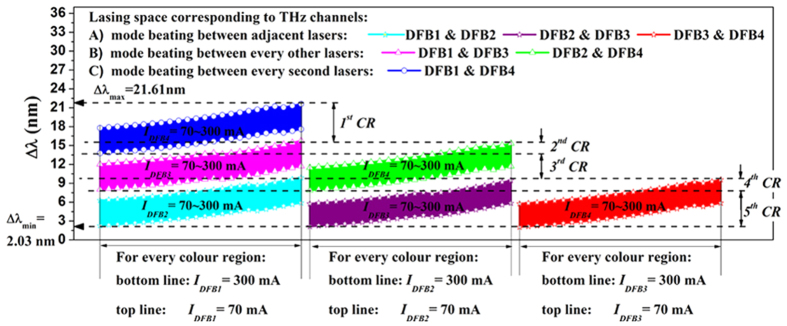
Continuous THz tuning range by the corresponding pair of DFB lasers demonstrating five channel regions (CRs). Tuning and wavelength difference between every possible pair of DFB lasers. Wavelength tuning in the *y*-axis results from varying the drive current of the first named laser of each pair in the legend; tuning in the x-axis corresponds to varying the current of the second laser of the pair.

In a complete system, the THz signals would be created by DFG between beams from pairs of DFB lasers. Despite the tuning ranges of these four DFBs being separated by several nanometers, the difference frequency can be tuned continuously, as is depicted in Fig. 3. The colored areas represent all the possible frequencies that can be realized by biasing the corresponding DFB currents. From the dashed lines in Fig. 3, we can separate the continuous THz tuning range into five channel regions (CRs) for discussion, which have different redundancy properties. CR1 (top, blue) in the Figure results from DFG between light from DFB1 and DFB4. As can be seen, there is no redundancy in this region, but for CR2 to CR5 there are always at least two combinations of diodes that can be used. In CR2, for example, the frequency band from 1.717 THz to 1.933 THz exhibits 3-fold redundancy between the blue, pink and green regions, which arise from Δλ_DFB4-DFB1_, Δλ_DFB3-DFB1_ and Δλ_DFB4-DFB2_, respectively. If DFB1 were to fail, this THz channel could be generated by using the corresponding frequencies from Δλ_DFB4-DFB2_, or, if DFB2 were to fail, this THz channel could be generated using Δλ_DFB4-DFB1_ or Δλ_DFB3-DFB1_. In other words, one alternative combination choice exists if DFB1 fails, and two alternatives appear if DFB2 fails. Furthermore, if DFB1 and DFB3 both fail, these THz channels could also be maintained by using DFG from Δλ_DFB4-DFB2_. Rich backup channel redundancy can thus be realized.

**Figure 4:**
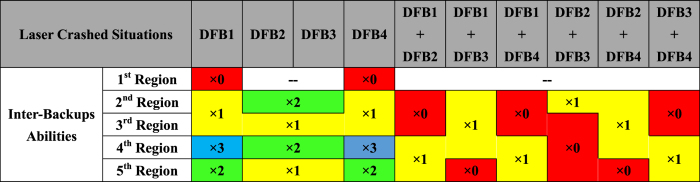
Redundancy properties of the PIC for THz frequency synthesis.

The redundancy arising from of every combination of lasers is presented in Fig. 4. The regions of the Figure colored red have no redundancy, while the yellow regions have one level of redundancy and the green regions have two. The regions of greatest reliability are colored blue, which have three levels of redundancy. The various backup properties of the PIC make it a reliable pumping source for THz wireless communication, especially in those circumstances that impose severe reliability specifications such as wireless communications between satellites in space where radiation might damage individual lasers.

Table 1 shows the tuning ranges that can be accessed using this THz pumping source. The range of continuous THz tunability extends from 0.254 THz to 2.723 THz, a range which covers almost the entire atmospheric THz communications window with low attenuation^3^. In calculating the THz frequency from the wavelength differences, we have made the approximation λ_1_ λ_2_ ≈ λ^2^ and taken λ = 1550 nm. This approximation introduces an error of <1%

**Figure 5:**
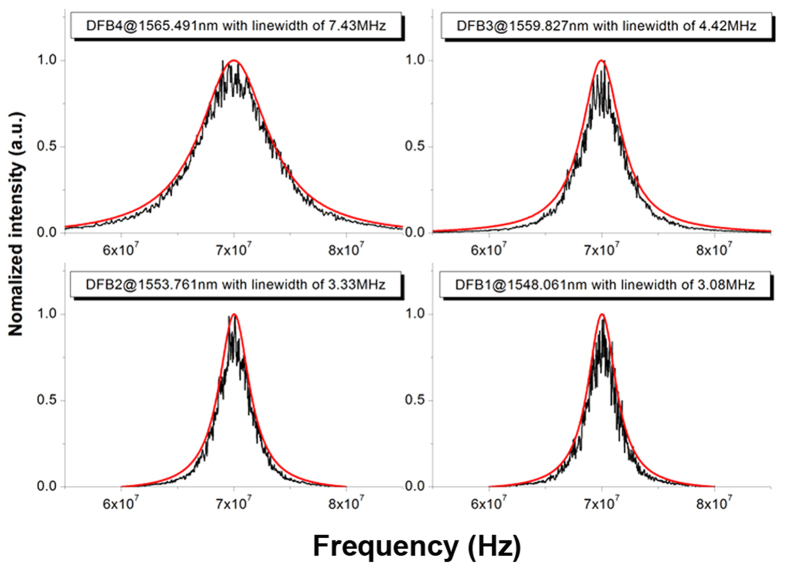
Measured optical linewidth of the four DFB lasers using the self-heterodyne method. Linewidth of the four DFB lasers with *I*_*SOA*_ = 100 mA and each of the DFBs biased at 100 mA. The black lines represent the measured data and the red curves have been fitted assuming a Lorentz profile.

The typical linewidth of each DFB is presented in Fig. 5, measured using the self-heterodyne method and taking light from the output port with the SOA biased at 100 mA. For each laser, the black curve shows the measured data and the red curve is a Lorentzian fit. The linewidth of three of the DFBs were measured to be around 3~4 MHz, which benefits from the relatively long cavity length of 1155 μm. The linewidth of the DFG signal would therefore be expected to be around 6~8 MHz. The linewidth of the fourth DFB was unexpectedly wide with a value of 7.43 MHz. This is believed to be the result of minor visible damage to the side wall gratings of the ridge waveguide.

**Figure 6:**
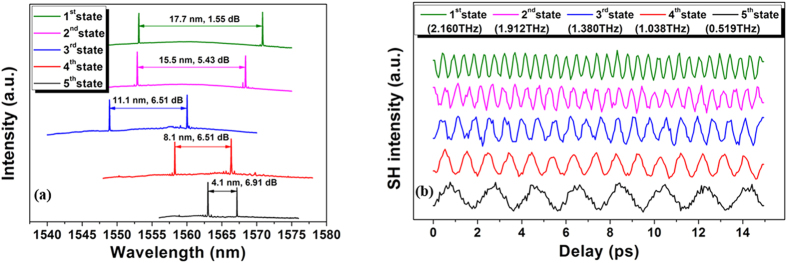
Typical optical spectra and SHG correlation traces for the five frequency range shown in Fig. 3. (**a**) Typical spectra for five frequency ranges of the PIC; the differences of peak wavelength and intensity between the peaks are shown. (**b**) Normalized SHG correlation traces measured simultaneously with the spectra shown in (**a**).

Figure 6 shows the mode-beating signals measured using an optical autocorrelator. The five normalized second-harmonic generation (SHG) curves in Fig. 6(b) correspond to the spectra with the same colors in Fig. 6(a), and these curves represent typical results for the five THz channel regions presented in Fig. 3. The clear DFG signal obtained by beating the light from two narrow linewidth DFBs results in a flat and standard sinusoidal SHG trace, which indicates a stable THz pumping source. The frequencies, corresponding to various THz channels, can be used as carrier frequencies for THz wireless communications.

**Figure 7:**
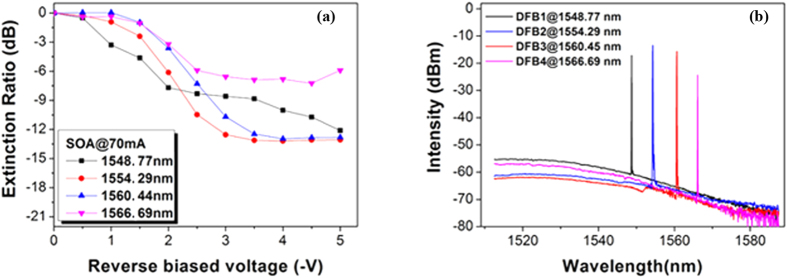
Measured ER from SOA side of the four EMLs and their operating optical spectrum. (**a**) ER of each EML tested from the coupled output port with SOA biased at 70 mA. (**b**) Each spectrum tested along with the ER test.

The extinction ratio (ER) performance of the electro-absorption modulated lasers (EMLs) is presented in Fig. 7. Optical spectra were recorded simultaneously with the ER test. Two of the four EMLs (for DFB2 and DFB3) have a relatively better ER of greater than −10 dB for a voltage swing of 2 V. The EAMs for DFB1 and DFB4 have ERs around −9 dB for a swing of 3.5 V and −7 dB for a swing of −3.5 V, respectively. The higher reverse bias voltages and reduced extinction ratios are the result of the relative differences between the EAM bandgap energy (which is the same for all of the devices) and the photon energy from the lasers (which is determined by the respective periods of the Bragg gratings). If the energy difference is small (lasing wavelength red-detuned by ≤30 nm), the insertion loss of the EAM is high and a larger voltage swing is required; equally when the energy difference is large (lasing wavelength red-detuned by ≥60 nm) the ER is reduced and a larger voltage swing is again required. The ER is limited by amplified spontaneous emission from the SOA. Because the loss associated with the 4 × 1 MMI coupler is 6 dB, using an arrayed waveguide grating (AWG) combiner instead would allow a shorter SOA to be used and so lead to an improvement in the direct current (DC) ER. Further optimization of the EAMs will be investigated in future designs.

**Figure 8:**
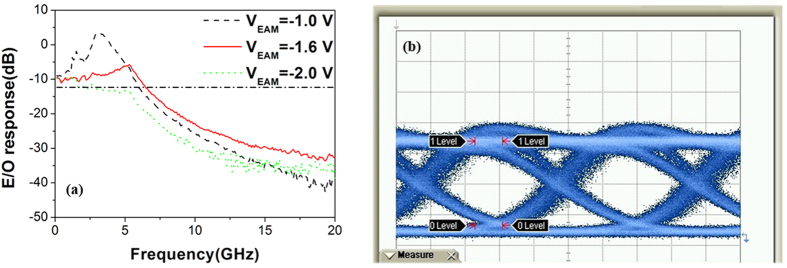
High speed characterization of the EAM associated with DFB4. (**a**) small signal E/O response at different DC biases *V*_*EAM*_. (**b**) eye diagram at *I*_*DFB*_ = 100 mA, *V*_*EAM*_  = −1.6 V under 5 Gb/s NRZ bit sequence modulation.

The RF performance for the DFB4 EAM is presented in Fig. 8, with the small signal frequency response shown in Fig. 8(a). The −3 dB bandwidth was ~6.5 GHz at an EAM bias of −1.6 V. The appearance of a resonance peak around the relaxation oscillation frequency and the anomalous trend of the bandwidth vs *V*_*EAM*_ in the E/O response of the EAM indicate that there is optical or electrical coupling between the EAM and the DFB laser^22^. Electrical coupling could originate from insufficient isolation between the laser and the EAM (measured to be ~2 kΩ)^22^ and could be resolved using proton implantation^23^. Optical coupling could originate from residual reflections from the MMI and/or SOA. The MMI is already a specifically designed to reduce back-reflections, with a 45° tilt at each corner of the input waveguides and the output waveguide; as a result the back reflections at the input waveguides of the MMI are expected to be reduced by at least 10 dB^24^. The dominant back-reflection is therefore expected to be from the SOA which could be suppressed effectively by optimizing the anti-reflection (AR)-coating. For higher modulation frequencies, straightforward design changes, such as deep-etching the EAM section to reduce the junction capacitance or using semi-insulating substrates to reduce stray capacitance, could be implemented^25^. Figure 8(b) shows the measured eye diagrams of the optical output at 5 Gbit/s for a non-return-to-zero (NRZ) pseudo-random-bit-sequence (PRBS) pulse pattern with a length of 2^31^−1, with a modulation voltage of 2 V_*pp*_ and a DC bias *V*_*EAM*_ = −1.6 V. The injection current in the DFB section was 100 mA. A clear open eye under 5 Gb/s modulation of the optical signal from the DFB was obtained. Additionally, the back-to-back performance demonstrated a bit error ratio (BER) of <10^−9^. Although these results cannot be extrapolated to predict the BER performance of a THz communication system with completely different noise statistics and limits, they do demonstrate the potential for on/off digital modulation of a THz carrier at data rates beyond 5 Gb/s. Results of THz wave generation and THz communication experiments will be reported out in due course.

### Summary

In conclusion, a PIC-based optoelectronic multi-frequency pumping source for THz communication systems has been demonstrated for the first time. The PICs were fabricated using simple side-wall gratings and QWI technologies, which eliminate the multiple stages of crystal regrowth required in traditional approaches and so reduce cost. The PIC produces up to four optical lines, i.e. up to three THz difference frequencies simultaneously. Using pairs of lasers, the frequency difference can be tuned continuously over the range from 0.254 to 2.723 THz. The THz frequency pumping source has a wide tuning range and good frequency stability, opening up applications such as THz wireless communications between CPUs, low-cost wireless communications for data centers and inter-satellite communications. The high level of channels backup redundancy makes the source particularly suitable for rigorous application environments, such as satellite to satellite communication or data centers. Along with developments in THz antennas, this PIC-based pumping source will be a significant enabler of THz wireless systems in the near future.

## Additional Information

**How to cite this article**: Xu, J. *et al*. Fully integrated multi-optoelectronic synthesizer for THz pumping source in wireless communications with rich backup redundancy and wide tuning range. *Sci. Rep.* **6**, 29084; doi: 10.1038/srep29084 (2016).

## Figures and Tables

**Table 1:**
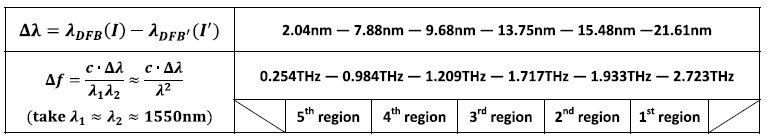
Numerical values of the wavelength differentials and beating frequencies in the five frequency bands.
